# Molecular Determinant of DIDS Analogs Targeting RAD51 Activity

**DOI:** 10.3390/molecules26185460

**Published:** 2021-09-08

**Authors:** Denis Velic, Alexandre Demeyer, Thibaut Peterlini, Houda Benhelli-Mokrani, Monique Mathé-Allainmat, Jean-Yves Masson, Fabrice Fleury

**Affiliations:** 1 Mechanism and Regulation of DNA Repair Team, UFIP, UMR 6286 CNRS, University of Nantes, F-44000 Nantes, France; velic.d@gmail.com (D.V.); alexandre.demeyer01@gmail.com (A.D.); houda.benhelli@univ-nantes.fr (H.B.-M.); 2 Genome Stability Laboratory, CHU de Québec Research Center, HDQ Pavilion, Oncology Division, 9 McMahon, Québec City, QC G1R 3S3, Canada; thibaut.peterlini@crchudequebec.ulaval.ca (T.P.); Jean-Yves.Masson@crchudequebec.ulaval.ca (J.-Y.M.); 3 Department of Molecular Biology, Medical Biochemistry, and Pathology, Laval University Cancer Research Center, Québec City, QC G1V 0A6, Canada; 4 CEISAM, UMR 6230 CNRS, University of Nantes, F-44000 Nantes, France; Monique.Mathe@univ-nantes.fr

**Keywords:** RAD51, recombinase activity, inhibitor, stilbene derivatives, cancer

## Abstract

RAD51 is the central protein in DNA repair by homologous recombination (HR), involved in several steps of this process. It is shown that overexpression of the RAD51 protein is correlated with increased survival of cancer cells to cancer treatments. For the past decade, RAD51 overexpression-mediated resistance has justified the development of targeted inhibitors. One of the first molecules described to inhibit RAD51 was the 4,4′-diisothiocyanato-stilbene-2,2′-disulfonic acid (DIDS) molecule. This small molecule is effective in inhibiting different functions of RAD51, however its mode of action and the chemical functions involved in this inhibition have not been identified. In this work, we used several commercial molecules derived from DIDS to characterize the structural determinants involved in modulating the activity of RAD51. By combining biochemical and biophysical approaches, we have shown that DIDS and two analogs were able to inhibit the binding of RAD51 to ssDNA and prevent the formation of D-loop by RAD51. Both isothiocyanate substituents of DIDS appear to be essential in the inhibition of RAD51. These results open the way to the synthesis of new molecules derived from DIDS that should be greater modulators of RAD51 and more efficient for HR inhibition.

## 1. Introduction

Cells are constantly subjected to endogenous and exogenous stress, both of them being able to affect the DNA integrity of its genome. Among the different deleterious DNA lesions, DNA double strand-breaks (DSB) are the most cytotoxic [1,2,3]. If these DSB are not repaired, the cell enters apoptosis leading to its death [4,5]. There are two main mechanisms able to repair those type of DNA alterations: non-homologous end-joining (NHEJ) and homologous recombination (HR). HR is an error free pathway that is preferentially activated during late S and G2 phases, whereas NHEJ is considered as an error prone mechanism active across the whole cell cycle [6,7,8]. Among all proteins involved in HR, RAD51 is the main one. RAD51 belongs to the recombinase family like RecA, DMC1, or RADA, all of these proteins showing similarities in sequences and structures [9,10]. RAD51 exhibits several essential activities during its DNA repair function, such as ssDNA and dsDNA binding, recombinase activity, homology searching, DNA strand invasion termed D-loop (displacement-loop), and ATP binding and hydrolysis [11,12].

RAD51 overexpression is commonly observed in various types of cancers [13], that leads to elevated HR efficiency and associated resistance to anti-cancer therapies [14]. In the last decade, RAD51 was described as an attractive therapeutic target whose inhibition is expected to improve the efficiency of oncogenic therapies. Indeed, after treatment-induced DSB, cells overexpressing RAD51 are able to survive [13,15]. Moreover, it was shown that the survival of cancer patients expressing higher levels of RAD51 is shorter and that a reduced amount of RAD51 in the cellular model, following antisense or ribozyme treatment, increases the effectiveness of cancer treatment by radiotherapy [16,17,18].

Different studies have highlighted new drugs targeting RAD51 [19], and the DIDS (4,4′-Diisothiocyano-2,2′-stilbenedisulfonic acid) molecule, a known chloride channel inhibitor, was one of the first molecules to be described to inhibit RAD51 functions [20].In the presence of DIDS, RAD51 becomes unable to bind ssDNA or dsDNA. Moreover, DIDS inhibits RAD51 recombinase activity by avoiding it to invade dsDNA and D-loop formation. Absence or presence of ATP has no influence on DIDS effect on RAD51.

Recently, DIDS cellular effect on RAD51 was confirmed in leukemia cells [21]. During maturation of B cells, a mechanism called switch class recombination needs AID (activation-induced cytidine deaminase) to perform correctly after infection by an antigen. Switch class recombination involves DNA recombination after induced DSB. [22]. Muramatsu et al. showed that DIDS is able to inhibit RAD51 foci formation and avoid B cells to use HR to repair DNA DSB. However, the mechanism used by DIDS to inhibit RAD51 remains unknown. To determine how DIDS could act against RAD51 activity, we used several DIDS analogs to assess their efficiency on various RAD51 activities and thus identify if these specific isothiocyanate functions of DIDS are determinant for the recombinase inhibition.

## 2. Results

### 2.1. Invasion Step of RAD51 Is Impaired by DIDS Derivatives

**Figure 1:**
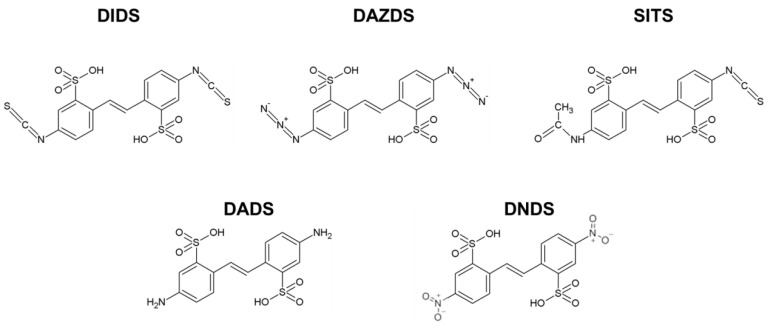
Chemical structures of DIDS characterized by two isothiocyanate groups and its derivatives DAZDS including two azido groups, SITS including isothiocyanate and acetamide group, DADS including two amino groups and DNDS including two nitro groups.

RAD51 has a pivotal role in the main steps of HR and in particular during synapsis where RAD51 nucleofilament searches for a homologous sequence in dsDNA and invades it to form the D-loop structure [23]. Using biochemical approaches, we assessed the effect of all selected/tested DIDS derivatives (Figure 1) on the D-loop structure formation.

**Figure 2:**
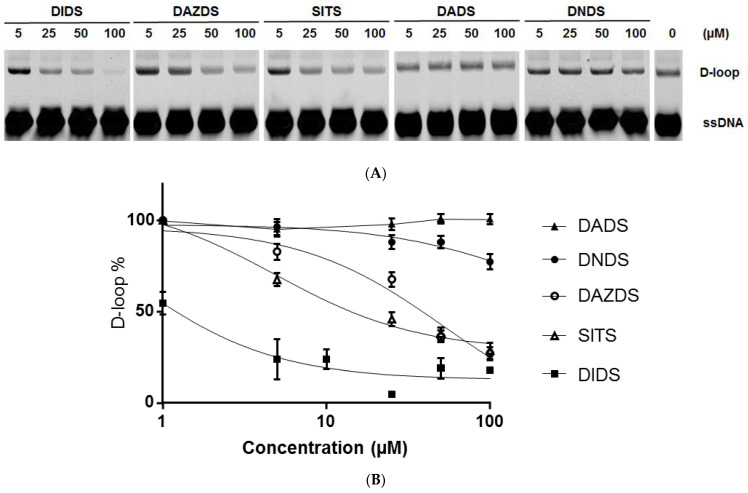
Effect of DIDS-derived small molecules on the ability to form D-loop structure. (**A**) D-loop structures in presence of increasing concentration of DIDS analogs (5, 25, 50, 145, and 100 µM) were separated and visualized on agarose gel. Last lane corresponds to the control condition without DIDS-derived molecule. (**B**) After quantification of signal intensity of shifted band corresponding to D-loop structure, the percent of D-loop is calculated in comparison to the condition in absence of drug and presented in relation to the increasing concentration (from 1 to 100 µM) of each small molecule. Three independent experiments were performed and each error bar represents s.d. statistical analysis.

Firstly, we incubated RAD51 with increasing concentrations of DIDS (from 1 to 100 µM) and then we calculated the percent of D-loop by quantifying the shifted band in comparison to the control condition in absence of DIDS (Figure 2A). As expected, DIDS inhibits D-loop intermediate structure in a dose-dependent manner, confirming the results already described in the literature [20]. The estimated IC50 of DIDS is 0.9 µM (Table 1).

Secondly, in the same approach we have tested the four other DIDS analogs on the D-loop formation by increasing their concentration until at 100 µM. Figure 2 provides evidence that there are two groups: the first group including DADS and DNDS has no effect, while the other group shows a significant inhibitory effect on the D-loop structure formation including DAZDS and SITS. From Figure 2B, IC50 of D-loop formation was determined and confirmed the previous observation since DAZDS and SITS have an IC50 of 41.3 µM and 29 µM, respectively, while DADS and DNDS molecules have an IC50 up to 100 µM (Table 1).

### 2.2. DIDS Analogs Modulate the RAD51 Nucleofilament Formation

**Figure 3:**
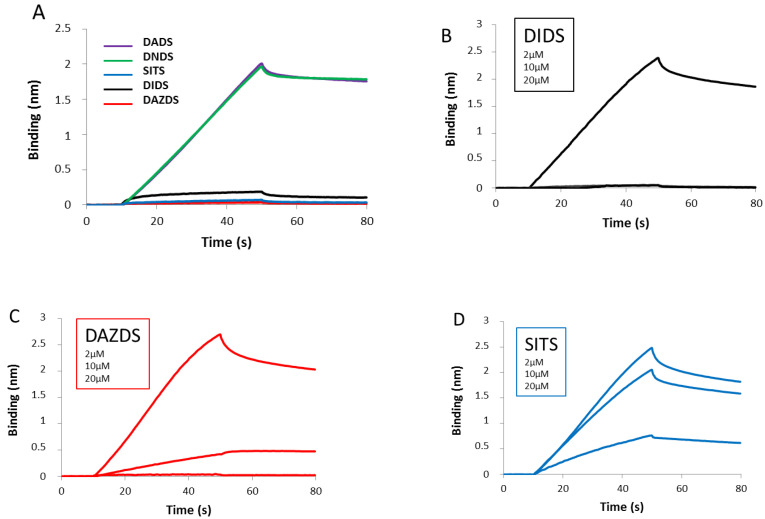

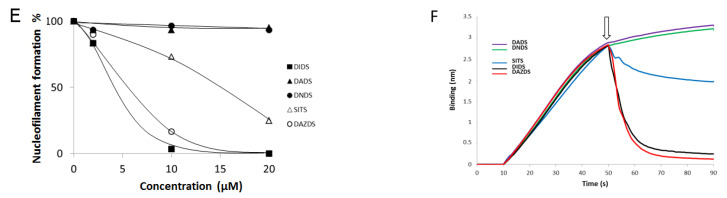
DIDS derivatives modulate the RAD51 nucleofilament formation. The association and dissociation between RAD51 and ssDNA were evaluated by Blitz interferometry. RAD51 (2 µM) was pre-incubated in phosphate buffer in absence or presence of each molecule at different concentrations before the measurement. The association phase followed until 50 s while the dissociation phase was monitored until 80 or 90 s, as indicated in graphs. (**A**) Effect of DIDS family on the ssDNA-RAD51 association was evaluated at 40 µM concentration of each molecule including DIDS, DADS, DAZDS, DNDS, and SITS. (**B**) DIDS, (**C**) DAZDS, and (**D**) SITS were incubated with RAD51 at increasing concentrations of 2, 10, and 20 µM for 5 min before the measurement by BLItz approach. (**E**) The percent of nucleofilament formation in presence of stilbene molecule was calculated in comparison to the condition in absence of drug after 50 s. This percentage was presented in function to the increasing concentration (from 2 to 20 µM) of each small molecule. (**F**) RAD51 associates with ssDNA until 50 s, and then each molecule is added at 40 µM as indicated by the narrow in the graph.

We have evaluated the steps preceding D-loop formation, namely the formation of the nucleofilament of RAD51. We investigated the effect of DIDS and its derivatives on ssDNA binding and the nucleofilament formation functions of RAD51 by bio-layer interferometry. Two approaches were undertaken: (i) to determine the impact of drugs on RAD51-ssDNA binding and (ii) to evaluate the ability of drugs to dissociate RAD51 nucleofilament. Concerning the first approach, we pre-incubated RAD51 with the drug of interest for 5 min before measuring ssDNA-RAD51 binding efficiency. Among the five drugs, three of them show an inhibitory effect on ssDNA-RAD51 association: DIDS, DAZDS, and SITS derivatives (Figure 3A). Furthermore, we investigated if that inhibitory effect is dose dependent. By pre-incubating RAD51 with three different concentrations (2, 10, and 20 µM) of each drug (DIDS, DAZDS, or SITS derivatives), we observed a dose-dependent effect of each molecule tested, suggesting a direct impact of the drug on RAD51, DIDS being the most efficient drug among them (Figure 3B–D). The Figure 3E shows the ability of the drugs to inhibit RAD51 nucleofilament formation as a function of the increasing concentration of each molecule. From 10 μM of DIDS, RAD51 nucleofilament formation is totally inhibited (Figure 3B). At a concentration of 20 μM, DAZDS inhibits the association of RAD51 with ssDNA by more than 80% (Figure 3C,E), while at the same concentration the SITS slightly reduces the formation of the nucleofilament by about 10–20% (Figure 3D,E).

Comparison of these three molecules suggests that DAZDS affects ssDNA RAD51 interactions in a similar way to DIDS, unlike SITS.

To this purpose, we incubated RAD51 with ssDNA, then we added the drug, and we measured the dissociation kinetic of RAD51 nucleofilament (Figure 3F). Interestingly, the three molecules inhibiting ssDNA-RAD51 association also destabilized the nucleofilament structure (Figure 3F). Among the five drugs analyzed, two of them, DIDS and DAZDS derivative, showed a strong effect on nucleofilament stability. They totally dissociated the structure and also avoided ssDNA-RAD51 association. SITS derivative showed a similar effect but with lower efficiency. The two last drugs, DADS and DNDS derivatives, were not able to dissociate the nucleofilament (Figure 3F).

### 2.3. Self-Association of RAD51 Is Sensitive to Presence of DIDS Derivatives

**Figure 4:**
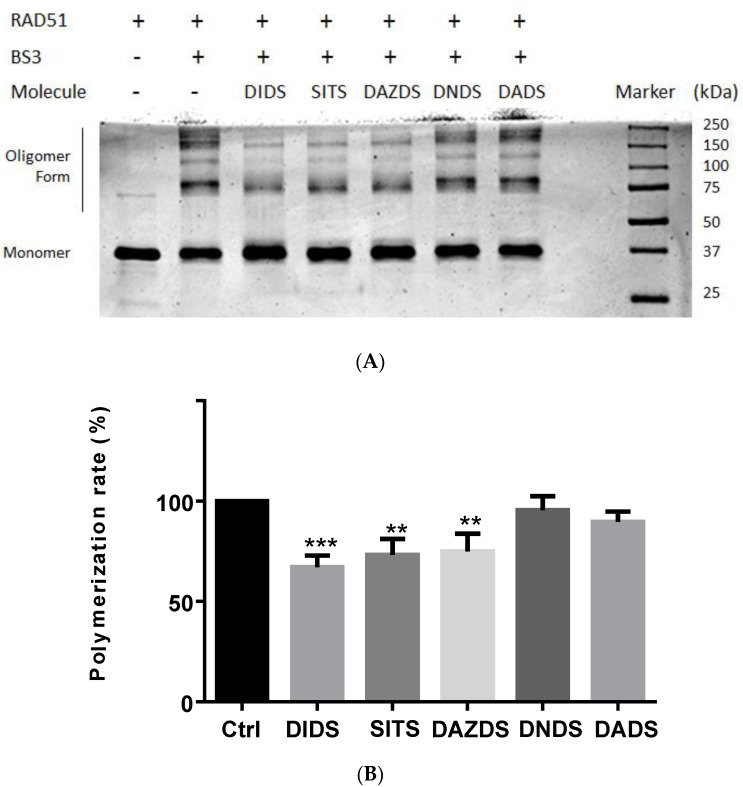
Self-association of RAD51 is inhibited in the presence of DIDS, SITS, and DAZDS. (**A**) The polymerization of RAD51 was estimated by cross-link approach (see Material and Method section). (**B**) Oligomer and monomer forms ratio is presented as a histogram graph. Three independent experiments were performed and statistical analysis was performed using paired Student’s *t*-test. Statistical significance was assumed at ** *p* < 0.01, *** *p* < 0.001. Each error bar represents s.d. statistical analysis.

The mechanism of action of these DIDS analogs molecules remains unclear, but they probably act on the protein–protein interaction site of RAD51. In order to assess their possible effect on the subunit–subunit RAD51 association, we investigated the oligomerization level of RAD51 in presence of these small molecules. By using BS3 cross-linker, we fixed the quaternary structure of RAD51 in absence or in presence of the five stilbene molecules. Oligomer and monomer forms were separated on SDS-PAGE and stained with Coomassie blue (Figure 4A). Oligomer forms include dimer, trimer, and high polymer while monomer form is well separated at 37 kDa. With a concentration of 50 µM of DNDS or DADS no effect on the migration profile was observed in comparison to control lane (RAD51 alone) suggesting no effect on the self-association of RAD51. On the opposite, the incubation with DIDS, SITS, and DAZDS at 50 µM induces a decrease of oligomer forms in favor of RAD51 monomer.

The quantification of the polymerization rate by calculating the oligomer/monomer form intensity ratio confirms this observation since DIDS, SITS, and DAZDS induced a significant decrease of polymerization rate at about 40% (Figure 4B) whereas DADS and DNDS have no effect.

## 3. Discussion

The research and development of new anti-cancer molecules constitutes a real challenge in the search for solutions to the treatment of cancer. The role of RAD51 in several types of cancer [13,24,25,26,27,28] has led many teams to develop inhibitors targeting this recombinase [29].

Among all these inhibitors, DIDS effect against RAD51 activity was demonstrated very early [20] but this molecule is relatively cytotoxic in fibroblast and prostatic cancer cells (data not shown), weakly specific, and was proved to be unstable in aqueous solution [30]. However, our strategy consisted of testing commercial DIDS derivatives to overcome these biological and chemical weaknesses and to better understand the mechanism of action of this molecule.

In this context, we assessed the impact of some analogs of DIDS on RAD51-mediated D-loop structure formation. We found three molecules where DIDS, DAZDS, and SITS are able to inhibit D-loop formation in a dose-dependent manner. Moreover, these three active molecules are also active in the binding of RAD51 to ssDNA. Indeed, the detection of the ssDNA-RAD51 interaction by bio-layer interferometry allowed to us to show that DIDS, as well as the DAZDS and SITS, analogs prevent the binding of RAD51 to ssDNA and dissociate the nucleofilament unlike the DADS and DNDS derivatives. This effect is concentration dependent and the DIDS molecule remains the most active among the three efficient one.

This result suggests that the isothiocyanate functions of DIDS, as well as the azido group of DAZDS, seem to be good bioisosteres, with electronic and steric properties in favor of a high potency to inhibit RAD51. This is confirmed with the weaker inhibitory effect of SITS, bearing only one isothiocyanate group, and pointing out the deleterious effect of an amide function in this 4-position of the stilbene moiety.

Moreover, the azido group is an inert function in biological systems [31] and, compared to isothiocyanate, is not potentially reactive with amines and other strong nucleophiles found in abundance in biological molecules. Indeed, two isothiocyanate substituents could react with the amino group of lysine of RAD51, which could be involved in the DNA interaction site of the protein. Ishida et al. have suggested that the DIDS compound inhibits the interaction between RAD51 and DNA [20]. On the other hand, this characteristic could explain the weak cytotoxicity of DAZDS in contrast to that of DIDS. It is interesting to note that therapeutic molecule containing an azido chemical group such as AZT (azidothymidine or 1-[(2R, 4S, 5S)-4-azido-5-(hydroxymethyl)oxolan-2-yl]-5-methylpyrimidine-2,4-dione[1]), were approved and marketed for the treatment of AIDS [32].

For the first time we demonstrate that other stilbene derivatives are able to act against RAD51 activity. These preliminary results should open interesting perspectives in the development of inhibitors of RAD51.

## 4. Material and Method

### 4.1. Chemical Compounds, Proteins Production and Purification

DIDS, SITS, DADS, DAZDS, and DNDS compounds were purchased from Sigma Aldrich (Saint-Louis, MO, USA). All molecules are solubilized in DMSO at 50 mM then diluted in phosphate buffer pH 7.4.

The cDNA of His-RAD51 was cloned into in the pET15a vector (Novagen). Human RAD51 WT was then over-expressed in Escherichia coli BL21-DE3 strain at 37 °C. RAD51-His proteins were purified on a NiNTA resin (Invitrogen, Thermo Fisher Scientific, Waltham, MA, USA). Imidazole used for purification was removed by dialysis. Protein concentrations were determined by BCA assay. The purity of proteins was analyzed by SDS-PAGE. RAD51 protein was kept in Tris-based storage buffer (20 mM Tris-HCl pH8; 200 mM KOAc pH8; 10% glycerol; 1 mM EDTA; 0.5 mM DTT; qsp water) at −80 °C prior to use [33].

### 4.2. Assessment of RAD51 Self-Association by Chemical Cross Linking

RAD51 at 8.5 µM was incubated with the indicated concentrations of chemical compounds for 5 min at room temperature in a buffer containing 20 mM sodium phosphate, 50 mM azidol, 1 mM MgCl2, and 1 mM ATP. The protein was cross-linked with 4.2 µM of BS3 (bis(sulfosuccinimidyl)suberate, Sigma) at room temperature for 30 min. Then the reaction was stopped by addition of Tris-HCl pH 8.1 at final concentration 33 mM for 15 min at room temperature. The sample was separated by SDS-PAGE at 10% and analyzed by Western blotting. Anti-RAD51 rabbit antibody was used as primary antibody (Sigma-Aldrich Cat# SAB2101936, RRID:AB_10607384), then secondary anti-rabbit antibody conjugated to alexafluor 680 (GAR 700: Molecular Probes Cat# A-21076, RRID:AB_2535736) was used for the detection. The quantification of monomer and cross-linked oligomers was performed by an Odyssey infrared imaging system scanner (LI-COR, Biosciences, Lincoln, NE, USA).

### 4.3. Supercoiled Plasmid DNA Production

Supercoiled plasmid production was described in Carbone et al., 2012 [34]. This method removed ssDNA. JM109 cells were transformed with pPB4.3 plasmid following protocol from the supplier (Stratagene, San Diego, CA, USA). The pUC19 plasmid was used as a positive control. After transformation, bacteria that grew on plates were picked up and incubated in LB media overnight at 37 °C under agitation (200 rpm). Plasmid DNA purification was held following steps of a plasmid DNA preparation kit from Qiagen.

### 4.4. D-Loop Assay

An IRD700 100 bp ssDNA probe (Integrated DNA Technologies, Leuven, Belgium) is used to assess D-loop formation activity of RAD51. Reactional buffer is prepared (25 mM Tris-HCl pH7.5; 1 mM DTT; 1 mM ATP; 1 mM CaCl2; qsp water) in the presence of 1 µM ssDNA probe. RAD51 is then added to the reaction, and the mixture is incubated for 5 min at 37 °C. Plasmid DNA (3 mM) is next added to the reaction for a final volume of 10 µL and incubated at 37 °C for 15 min. Reaction is deproteinized by adding stop buffer (20 mM Tris-HCl pH7.5; 5 mg/mL proteinase K; qsp water) and the mixture is incubated for 20 min at 37 °C. After adding gel loading dye, the samples are separated on an 1% agarose gel and results are revealed on a LICOR scanner (Odyssey Imager, LI-COR, Biosciences, Lincoln, NE, USA).

### 4.5. DNA Binding Assay

PolydT DNA (1 µM) is biotinylated to allow its binding to the streptavidin-coated biosensor. Two methods of evaluation are used: the former by pre-incubating RAD51 with the drug to test and the latter by adding the drug after RAD51 is bound to DNA. In the first case, 2 µM RAD51 is incubated with the drug for 10 min on ice, then RAD51-ssDNA binding measurement is performed (baseline phase: 10 s; association phase: 40 s; dissociation phase: 30 s). In the second case, RAD51-ssDNA binding is analyzed, and after the association phase, the drug is added to the reaction and dissociation effect is measured (baseline phase: 10 s; association phase: 50 s; dissociation phase: 40 s). For the two cases, reaction buffer is composed of Phosphate Buffer saline (PBS) and 1 mM ATP. The tip is regenerated with 50 mM NaOH during 40 s before reuse. Measures are realized on a BLItz device from ForteBio.

## 5. Conclusions

Over the past decade, the research and development of new molecules targeting RAD51 has been intensified considerably. To minimize the toxic effects of DIDS and its instability, it is necessary to identify novel inhibitors more specific to RAD51.

Our study showed that the DAZDS molecule (azido analog of DIDS) was able to inhibit RAD51 activity in vitro. These results open up promising perspectives in the development of new molecules derived from stilbene bis- for chemo and radio-sensitization of tumors to conventional anti-cancer treatments.

## Figures and Tables

**Table 1:** IC50 determination from D-loop data of the Figure 2.

Molecules	DADS	DNDS	DIDS	DAZDS	SITS
IC50 (µM)	>100	>100	0.9	41.3	29

## Data Availability

The data presented in this study are available on request from the corresponding author.

## Sample Availability

Samples of the compounds are not available from the authors.

## Abbreviations

DIDS	4,4′-diisothiocyanatostilbene-2,2′-disulfonic acid;
DADS	4,4′-diaminostilbene-2,2′-disulfonic acid;
DAZDS	4,4′-diazidostilbene-2,2′-disulfonic acid;
DNDS	4,4′-dinitrostilbene-2,2′-disulfonic acid;
SITS	4-acetamido-4′-isothiocyanatostilbene-2,2′-disulfonic acid;
AZT	(azidothymidine or 1-[(2R, 4S, 5S)-4-azido-5-(hydroxymethyl)oxolan-2-yl]-5-methylpyrimidine-2,4-dione;
HR	Homologous recombination;
NHEJ	Non-homologous end-joining;
PBS	Phosphate buffer saline;
BS3	bis-sulfosuccinimidyl suberate;
GAR 700	Goat anti-rabbit antibody conjugated to AlexaFluor 700 nm;
BLItz	Bio-layer interferometry
